# 1,1′′-Bis(prop-2-en-1-yl)-1,1′′,2,2′′-tetra­hydro­dispiro­[indole-3,7′-[6,9]diaza­tricyclo­[7.3.0.0^2,6^]dodecane-8′,3′′-indole]-2,2′′-dione

**DOI:** 10.1107/S1600536812019216

**Published:** 2012-05-05

**Authors:** Khalil Al Mamari, Hamid Ennajih, Rachid Bouhfid, El Mokhtar Essassi, Seik Weng Ng

**Affiliations:** a Laboratoire de Chimie Organique Hétérocyclique, Pôle de Compétences Pharmacochimie, Université Mohammed V–Agdal, BP 1014 Avenue Ibn Batout, Rabat, Morocco; b Institute of Nanomaterials and Nanotechnology MAScIR, Avenue de l’Armée Royale, Rabat, Morocco; c Department of Chemistry, University of Malaya, 50603 Kuala Lumpur, Malaysia; d Chemistry Department, King Abdulaziz University, PO Box 80203 Jeddah, Saudi Arabia

## Abstract

The mol­ecule of the title compound, C_30_H_32_N_4_O_2_, lies on a twofold rotation axis that passes through the mid-points of the C—C bonds of the piperazine ring, which adopts a chair conformation. The pyrrolidine ring that is fused to the piperazine ring adopts an envelope conformation (in which the N atom represents the flap). The indoline fused-ring system is nearly planar (r.m.s. deviation = 0.044 Å); the two symmetry-related indoline fused-rings systems are aligned at 71.44 (3)°.

## Related literature

For background to the class of dispiro compounds, see: Al Mamari *et al.* (2012 ▶). For a related structure, see: Sugaleshini *et al.* (2006 ▶).
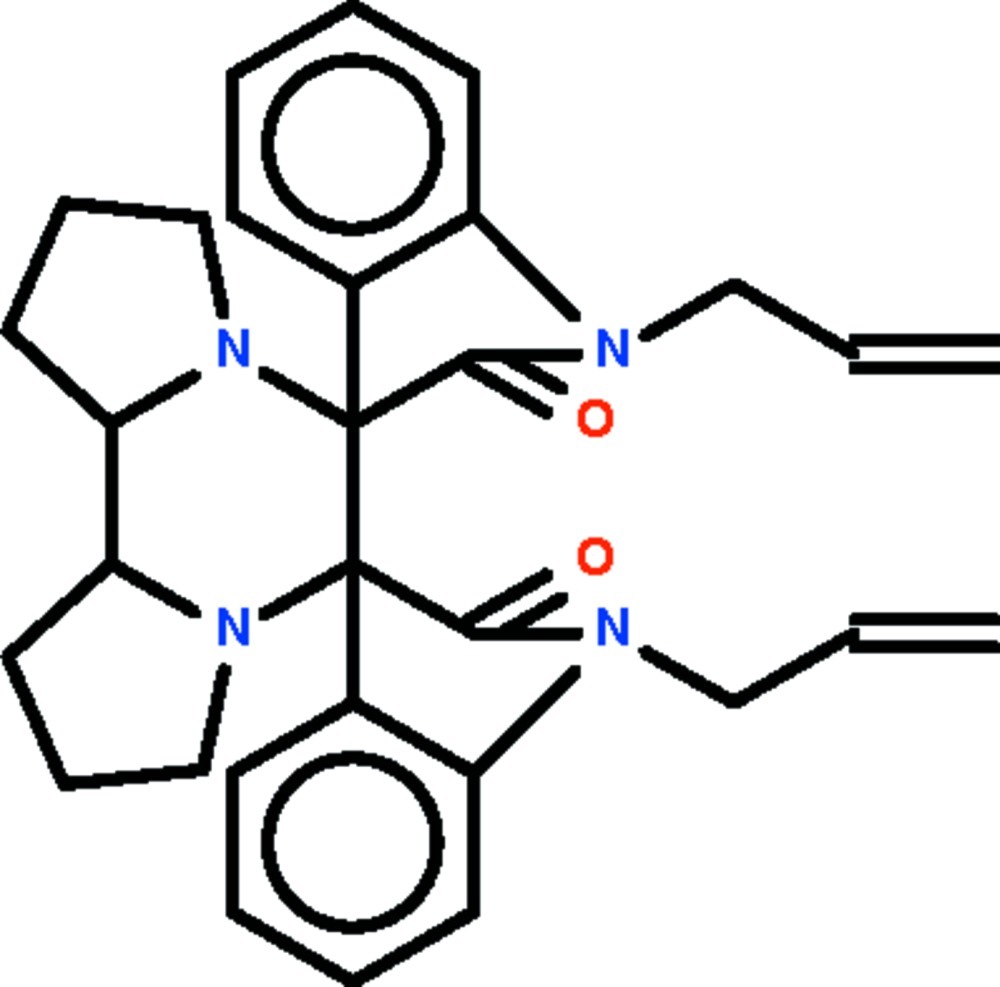


## Experimental

### 

#### Crystal data

C_30_H_32_N_4_O_2_
*M* *_r_* = 480.60
Monoclinic, 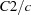

*a* = 14.9484 (2) Å
*b* = 9.9173 (1) Å
*c* = 17.5713 (3) Å
β = 111.119 (1)°
*V* = 2429.94 (6) Å^3^
*Z* = 4
Mo *K*α radiation
μ = 0.08 mm^−1^
*T* = 293 K
0.18 × 0.16 × 0.14 mm

#### Data collection

Bruker APEX DUO diffractometer
Absorption correction: multi-scan (*SADABS*; Sheldrick, 1996 ▶) *T*_min_ = 0.985, *T*_max_ = 0.988
13297 measured reflections
2797 independent reflections
2441 reflections with *I* > 2σ(*I*)
*R*_int_ = 0.024

#### Refinement

*R*[*F* ^2^ > 2σ(*F* ^2^)] = 0.040
*wR*(*F* ^2^) = 0.119
*S* = 1.00
2797 reflections
163 parameters
H-atom parameters constrained
Δρ_max_ = 0.27 e Å^−3^
Δρ_min_ = −0.18 e Å^−3^

### 

Data collection: *APEX2* (Bruker, 2010 ▶); cell refinement: *SAINT* (Bruker, 2010 ▶); data reduction: *SAINT*; program(s) used to solve structure: *SHELXS97* (Sheldrick, 2008 ▶); program(s) used to refine structure: *SHELXL97* (Sheldrick, 2008 ▶); molecular graphics: *X-SEED* (Barbour, 2001 ▶); software used to prepare material for publication: *publCIF* (Westrip, 2010 ▶).

## Supplementary Material

Crystal structure: contains datablock(s) global, I. DOI: 10.1107/S1600536812019216/xu5527sup1.cif

Structure factors: contains datablock(s) I. DOI: 10.1107/S1600536812019216/xu5527Isup2.hkl

Additional supplementary materials:  crystallographic information; 3D view; checkCIF report

## Figures and Tables

**Fig. 1.**
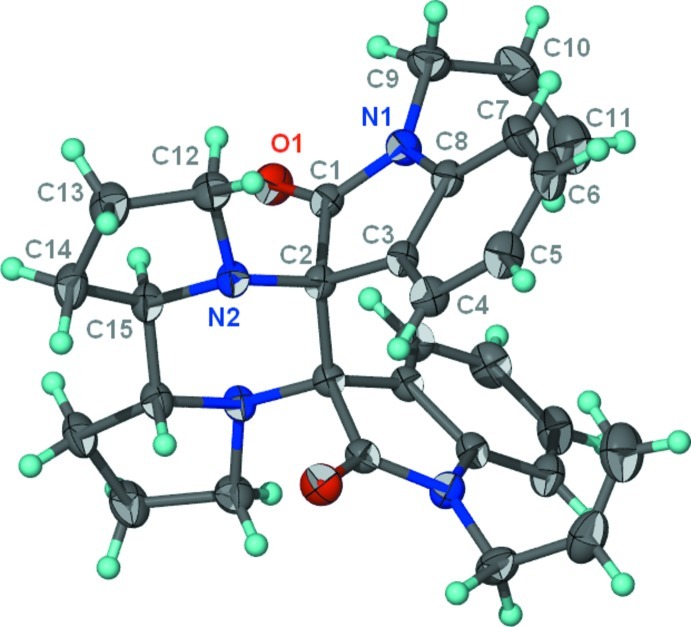
Thermal ellipsoid plot (Barbour, 2001) of C30H32N4O2 at the 50% probability level. Symmetry-related atoms are not labeled.

## supplementary crystallographic information

### Comment

We reported the the 1,3-dipolar cycloaddition of 1-allyl-5-haloisatin derivatives as dipolarophiles with the azomethine ylides generated *in situ* from *N*-allylisatin and L-proline to yield dispiro-oxindoles (Al Mamari *et al.*, 2012). Although one of the reactants is optically active, the product crystallizes in a centrosymmetric space group, as does the unsubstituted compound, C_30_H_32_N_4_O_20 _(Scheme I), which represents the homolog of this class of compounds. The molecule lies on a two-fold rotation axis that passes through the two mid-points of the C–C bonds of the piperazine ring, which adopts a chair conformation. The pyrrolidine ring that is fused to the piperazine ring adopts an envelope conformation (in which the N atom represents the flap) (Fig. 1). The indoline fused-ring system is planar (r.m.s. deviation 0.044 Å); the two fused-rings are aligned at 71.44 (3) °.

The reaction of acenaphthracenequinone and L-proline gave acenapthene-2-spiro-5'-perhydrodipyrrolo[1,2-*a*;2',1'-*c*]pyrazine-6'-spiro-2''-acenapthene-1,1''-dione, which also crystallizes in a centrosymmetric space group (Sugaleshini *et al.*, 2006).

### Experimental

A mixture of 1-allyl-indoline-2,3-dione (1 g, 0.005 mol) and proline (0.5 g, 0.004 mole) in ethanol (20 ml) was heated for 2 hours. On completion of the reaction as indicated by TLC, water (50 ml) was added. The precipitate was collected and recrystallized from ethanol to yield colorless crystals.

### Refinement

The aromatic and methylene H-atoms were placed in calculated positions (C–H 0.93–0.97 Å) and were included in the refinement in the riding model approximation, with *U*(H) set to 1.2*U*(C).

### Crystal data

**Table:**

C_30_H_32_N_4_O_2_	*F*(000) = 1024
*M**_r_* = 480.60	*D*_x_ = 1.314 Mg m^−^^3^
Monoclinic, *C*2/*c*	Mo *K*α radiation, λ = 0.71073 Å
Hall symbol: -C 2yc	Cell parameters from 8483 reflections
*a* = 14.9484 (2) Å	θ = 2.5–32.7°
*b* = 9.9173 (1) Å	µ = 0.08 mm^−^^1^
*c* = 17.5713 (3) Å	*T* = 293 K
β = 111.119 (1)°	Prism, colorless
*V* = 2429.94 (6) Å^3^	0.18 × 0.16 × 0.14 mm
*Z* = 4	

### Data collection

**Table:**

Bruker APEX DUO diffractometer	2797 independent reflections
Radiation source: fine-focus sealed tube	2441 reflections with *I* > 2σ(*I*)
Graphite monochromator	*R*_int_ = 0.024
ω scans	θ_max_ = 27.5°, θ_min_ = 2.5°
Absorption correction: multi-scan (*SADABS*; Sheldrick, 1996)	*h* = −11→19
*T*_min_ = 0.985, *T*_max_ = 0.988	*k* = −12→12
13297 measured reflections	*l* = −22→20

### Refinement

**Table:**

Refinement on *F*^2^	Primary atom site location: structure-invariant direct methods
Least-squares matrix: full	Secondary atom site location: difference Fourier map
*R*[*F*^2^ > 2σ(*F*^2^)] = 0.040	Hydrogen site location: inferred from neighbouring sites
*wR*(*F*^2^) = 0.119	H-atom parameters constrained
*S* = 1.00	*w* = 1/[σ^2^(*F*_o_^2^) + (0.0678*P*)^2^ + 1.2982*P*] where *P* = (*F*_o_^2^ + 2*F*_c_^2^)/3
2797 reflections	(Δ/σ)_max_ = 0.001
163 parameters	Δρ_max_ = 0.27 e Å^−^^3^
0 restraints	Δρ_min_ = −0.18 e Å^−^^3^

### Fractional atomic coordinates and isotropic or equivalent isotropic displacement parameters (Å^2^)

**Table:**

	*x*	*y*	*z*	*U*_iso_*/*U*_eq_	
O1	0.43126 (6)	0.47958 (9)	0.37259 (6)	0.0390 (2)	
N1	0.52761 (7)	0.66607 (10)	0.39788 (6)	0.0315 (2)	
N2	0.59105 (6)	0.39146 (9)	0.30977 (6)	0.0285 (2)	
C1	0.49026 (7)	0.54901 (11)	0.35787 (6)	0.0283 (2)	
C2	0.53923 (7)	0.51947 (10)	0.29458 (6)	0.0255 (2)	
C3	0.61013 (7)	0.63591 (11)	0.31150 (7)	0.0272 (2)	
C4	0.68373 (8)	0.66113 (12)	0.28314 (8)	0.0348 (3)	
H4	0.6923	0.6064	0.2433	0.042*	
C5	0.74493 (8)	0.76994 (13)	0.31530 (9)	0.0406 (3)	
H5	0.7937	0.7891	0.2958	0.049*	
C6	0.73379 (9)	0.84936 (13)	0.37577 (9)	0.0409 (3)	
H6	0.7752	0.9215	0.3964	0.049*	
C7	0.66169 (9)	0.82346 (12)	0.40655 (8)	0.0373 (3)	
H7	0.6546	0.8762	0.4478	0.045*	
C8	0.60104 (8)	0.71628 (11)	0.37335 (7)	0.0289 (2)	
C9	0.50268 (10)	0.72080 (14)	0.46422 (8)	0.0419 (3)	
H9A	0.5611	0.7350	0.5110	0.050*	
H9B	0.4644	0.6548	0.4797	0.050*	
C10	0.44850 (11)	0.85017 (16)	0.44444 (10)	0.0520 (4)	
H10	0.4341	0.8903	0.4865	0.062*	
C11	0.41912 (12)	0.91320 (17)	0.37480 (12)	0.0618 (4)	
H11A	0.4316	0.8775	0.3307	0.074*	
H11B	0.3857	0.9939	0.3691	0.074*	
C12	0.66448 (9)	0.37194 (13)	0.39119 (7)	0.0376 (3)	
H12A	0.6392	0.3916	0.4337	0.045*	
H12B	0.7200	0.4284	0.3989	0.045*	
C13	0.68964 (10)	0.22205 (14)	0.39088 (9)	0.0486 (4)	
H13A	0.7495	0.2111	0.3819	0.058*	
H13B	0.6959	0.1804	0.4425	0.058*	
C14	0.60698 (9)	0.15842 (13)	0.32126 (9)	0.0424 (3)	
H14A	0.6275	0.1318	0.2770	0.051*	
H14B	0.5822	0.0799	0.3401	0.051*	
C15	0.53166 (8)	0.26936 (11)	0.29411 (7)	0.0315 (3)	
H15	0.4925	0.2690	0.3284	0.038*	

### Atomic displacement parameters (Å^2^)

**Table:**

	*U*^11^	*U*^22^	*U*^33^	*U*^12^	*U*^13^	*U*^23^
O1	0.0364 (4)	0.0421 (5)	0.0445 (5)	−0.0111 (4)	0.0218 (4)	0.0000 (4)
N1	0.0325 (5)	0.0313 (5)	0.0345 (5)	−0.0039 (4)	0.0167 (4)	−0.0031 (4)
N2	0.0229 (4)	0.0263 (5)	0.0318 (5)	0.0005 (3)	0.0046 (4)	0.0013 (4)
C1	0.0253 (5)	0.0298 (5)	0.0297 (5)	−0.0008 (4)	0.0099 (4)	0.0028 (4)
C2	0.0213 (5)	0.0263 (5)	0.0293 (5)	−0.0012 (4)	0.0095 (4)	0.0006 (4)
C3	0.0222 (5)	0.0264 (5)	0.0315 (5)	−0.0012 (4)	0.0078 (4)	0.0021 (4)
C4	0.0276 (5)	0.0384 (6)	0.0411 (6)	−0.0026 (5)	0.0156 (5)	−0.0004 (5)
C5	0.0270 (6)	0.0423 (7)	0.0547 (7)	−0.0064 (5)	0.0173 (5)	0.0041 (6)
C6	0.0289 (6)	0.0313 (6)	0.0579 (8)	−0.0081 (5)	0.0101 (5)	−0.0005 (5)
C7	0.0345 (6)	0.0309 (6)	0.0443 (7)	−0.0042 (5)	0.0116 (5)	−0.0060 (5)
C8	0.0253 (5)	0.0271 (5)	0.0337 (5)	−0.0005 (4)	0.0099 (4)	0.0027 (4)
C9	0.0484 (7)	0.0470 (7)	0.0361 (6)	−0.0057 (6)	0.0223 (6)	−0.0062 (5)
C10	0.0529 (8)	0.0475 (8)	0.0669 (9)	−0.0053 (6)	0.0353 (7)	−0.0185 (7)
C11	0.0509 (9)	0.0482 (9)	0.0854 (12)	0.0068 (7)	0.0234 (9)	−0.0022 (8)
C12	0.0302 (6)	0.0380 (6)	0.0362 (6)	0.0025 (5)	0.0017 (5)	0.0024 (5)
C13	0.0389 (7)	0.0392 (7)	0.0537 (8)	0.0078 (5)	−0.0004 (6)	0.0065 (6)
C14	0.0386 (7)	0.0292 (6)	0.0515 (7)	0.0049 (5)	0.0066 (6)	0.0061 (5)
C15	0.0281 (5)	0.0261 (5)	0.0375 (6)	−0.0008 (4)	0.0085 (5)	0.0022 (4)

### Geometric parameters (Å, º)

**Table:**

O1—C1	1.2178 (13)	C7—H7	0.9300
N1—C1	1.3677 (15)	C9—C10	1.490 (2)
N1—C8	1.4070 (14)	C9—H9A	0.9700
N1—C9	1.4511 (15)	C9—H9B	0.9700
N2—C2	1.4608 (13)	C10—C11	1.301 (3)
N2—C15	1.4677 (14)	C10—H10	0.9300
N2—C12	1.4688 (14)	C11—H11A	0.9300
C1—C2	1.5637 (14)	C11—H11B	0.9300
C2—C3	1.5226 (14)	C12—C13	1.5339 (18)
C2—C2^i^	1.583 (2)	C12—H12A	0.9700
C3—C4	1.3843 (15)	C12—H12B	0.9700
C3—C8	1.3933 (16)	C13—C14	1.5273 (19)
C4—C5	1.3958 (17)	C13—H13A	0.9700
C4—H4	0.9300	C13—H13B	0.9700
C5—C6	1.380 (2)	C14—C15	1.5226 (16)
C5—H5	0.9300	C14—H14A	0.9700
C6—C7	1.3929 (18)	C14—H14B	0.9700
C6—H6	0.9300	C15—C15^i^	1.497 (2)
C7—C8	1.3824 (16)	C15—H15	0.9800
			
C1—N1—C8	111.22 (9)	C10—C9—H9A	108.7
C1—N1—C9	123.71 (10)	N1—C9—H9B	108.7
C8—N1—C9	124.63 (10)	C10—C9—H9B	108.7
C2—N2—C15	115.95 (8)	H9A—C9—H9B	107.6
C2—N2—C12	116.96 (9)	C11—C10—C9	127.15 (14)
C15—N2—C12	105.26 (9)	C11—C10—H10	116.4
O1—C1—N1	124.30 (10)	C9—C10—H10	116.4
O1—C1—C2	127.27 (10)	C10—C11—H11A	120.0
N1—C1—C2	108.39 (9)	C10—C11—H11B	120.0
N2—C2—C3	109.72 (8)	H11A—C11—H11B	120.0
N2—C2—C1	112.72 (8)	N2—C12—C13	102.86 (10)
C3—C2—C1	100.98 (8)	N2—C12—H12A	111.2
N2—C2—C2^i^	109.62 (6)	C13—C12—H12A	111.2
C3—C2—C2^i^	114.12 (7)	N2—C12—H12B	111.2
C1—C2—C2^i^	109.52 (9)	C13—C12—H12B	111.2
C4—C3—C8	119.41 (10)	H12A—C12—H12B	109.1
C4—C3—C2	130.87 (10)	C14—C13—C12	105.93 (10)
C8—C3—C2	109.23 (9)	C14—C13—H13A	110.5
C3—C4—C5	118.94 (11)	C12—C13—H13A	110.5
C3—C4—H4	120.5	C14—C13—H13B	110.5
C5—C4—H4	120.5	C12—C13—H13B	110.5
C6—C5—C4	120.64 (11)	H13A—C13—H13B	108.7
C6—C5—H5	119.7	C15—C14—C13	104.24 (10)
C4—C5—H5	119.7	C15—C14—H14A	110.9
C5—C6—C7	121.24 (11)	C13—C14—H14A	110.9
C5—C6—H6	119.4	C15—C14—H14B	110.9
C7—C6—H6	119.4	C13—C14—H14B	110.9
C8—C7—C6	117.35 (12)	H14A—C14—H14B	108.9
C8—C7—H7	121.3	N2—C15—C15^i^	107.95 (8)
C6—C7—H7	121.3	N2—C15—C14	102.03 (9)
C7—C8—C3	122.38 (11)	C15^i^—C15—C14	116.41 (10)
C7—C8—N1	127.46 (11)	N2—C15—H15	110.0
C3—C8—N1	110.04 (9)	C15^i^—C15—H15	110.0
N1—C9—C10	114.21 (11)	C14—C15—H15	110.0
N1—C9—H9A	108.7		
			
C8—N1—C1—O1	173.93 (11)	C4—C5—C6—C7	0.1 (2)
C9—N1—C1—O1	1.24 (18)	C5—C6—C7—C8	−0.85 (19)
C8—N1—C1—C2	−3.80 (12)	C6—C7—C8—C3	−0.04 (18)
C9—N1—C1—C2	−176.49 (10)	C6—C7—C8—N1	175.65 (11)
C15—N2—C2—C3	−178.61 (9)	C4—C3—C8—C7	1.66 (17)
C12—N2—C2—C3	56.22 (12)	C2—C3—C8—C7	174.50 (10)
C15—N2—C2—C1	69.71 (11)	C4—C3—C8—N1	−174.70 (10)
C12—N2—C2—C1	−55.46 (12)	C2—C3—C8—N1	−1.86 (12)
C15—N2—C2—C2^i^	−52.56 (13)	C1—N1—C8—C7	−172.47 (11)
C12—N2—C2—C2^i^	−177.72 (10)	C9—N1—C8—C7	0.14 (19)
O1—C1—C2—N2	−58.18 (14)	C1—N1—C8—C3	3.66 (13)
N1—C1—C2—N2	119.46 (10)	C9—N1—C8—C3	176.27 (11)
O1—C1—C2—C3	−175.17 (11)	C1—N1—C9—C10	−113.01 (14)
N1—C1—C2—C3	2.47 (11)	C8—N1—C9—C10	75.28 (15)
O1—C1—C2—C2^i^	64.14 (12)	N1—C9—C10—C11	3.0 (2)
N1—C1—C2—C2^i^	−118.22 (7)	C2—N2—C12—C13	169.82 (10)
N2—C2—C3—C4	52.23 (15)	C15—N2—C12—C13	39.45 (12)
C1—C2—C3—C4	171.40 (11)	N2—C12—C13—C14	−17.94 (15)
C2^i^—C2—C3—C4	−71.23 (15)	C12—C13—C14—C15	−8.73 (15)
N2—C2—C3—C8	−119.51 (10)	C2—N2—C15—C15^i^	60.60 (14)
C1—C2—C3—C8	−0.34 (11)	C12—N2—C15—C15^i^	−168.44 (11)
C2^i^—C2—C3—C8	117.03 (11)	C2—N2—C15—C14	−176.24 (10)
C8—C3—C4—C5	−2.36 (17)	C12—N2—C15—C14	−45.28 (12)
C2—C3—C4—C5	−173.40 (11)	C13—C14—C15—N2	32.17 (13)
C3—C4—C5—C6	1.51 (19)	C13—C14—C15—C15^i^	149.40 (12)

Symmetry code: (i) −*x*+1, *y*, −*z*+1/2.
